# Flow Injection Analysis of Mercury Using 4-(Dimethylamino) Benzaldehyde-4-Ethylthiosemicarbazone as the Ionophore of a Coated Wire Electrode

**DOI:** 10.3390/s121114968

**Published:** 2012-11-06

**Authors:** Tara F. Tahir, Abdussalam Salhin, Sulaiman Ab Ghani

**Affiliations:** Pusat Pengajian Sains Kimia, Universiti Sains Malaysia, 11800 USM P. Pinang, Malaysia; E-Mails: taratahir73@yahoo.com (T.F.T.); abdussalam@usm.my (A.S.)

**Keywords:** coated wire electrode, 4-(dimethylamino) benzaldehyde-4-ethylthiosemicarbazone, mercury(II) ion-selective electrode, flow injection analysis

## Abstract

A flow injection analysis (FIA) incorporating a thiosemicarbazone-based coated wire electrode (CWE) was developed method for the determination of mercury(II). A 0.1 M KNO_3_ carrier stream with pH between 1 and 5 and flow rate of 1 mL·min^−1^ were used as optimum parameters. A linear plot within the concentration range of 5 × 10^−6^–0.1 M Hg(II), slope of 27.8 ± 1 mV per decade and correlation coefficient (R^2^) of 0.984 were obtained. The system was successfully applied for the determination of mercury(II) in dental amalgam solutions and spiked environmental water samples. Highly reproducible measurements with relative standard deviation (RSD < 1% (n = 3)) were obtained, giving a typical throughput of 30 samples·h^−1^.

## 1. Introduction

Among the heavy metal ions found in environmental waters mercury(II) is one of the most hazardous. It can be taken up by plankton, which is then consumed by fish and finally reach humans, the last destination in the food chain, or be ingested through polluted drinking water. The permissible mercury(II) level in the drinking water set by the WHO is 1 μg·L^−1^ [1]. Mercury(II) is essentially nephrotoxic [2]. It has been claimed to inhibit or deactivate the biological functions of several enzymes by binding to the sulfhydryl group of the enzyme [3,4].

Several instrumental methods such as atomic fluorescence spectrometry (AFS) [5], cold vapor atomic absorption spectroscopy (AAS) [6], inductively coupled plasma (ICP) [7], and X-ray fluorescence (XRF) [8] have been applied for determination of mercury(II). These methods are not cost effective, are labour intensive and not practical for *in situ* or on site analyses. Analysis by chemical ion sensors or ion selective electrodes (ISEs), on the other hand, provides an alternative to those mentioned for quite the opposite reasons but offering equal performance. The key advantages of these electrodes are their cost and ease of manufacture, coupled with analytical performances comparable to the state of the art instrumentation mentioned earlier. Ironically, mercury(II) ion sensors are quite rare in the marketplace, although over the years reports on the development of such electrodes have been very encouraging. Several mercury(II) ion sensors based on heterogeneous membranes, consisting of binder polymers like polyvinylchloride (PVC), polystyrene and epoxy resin (Araldite) have been used with ion-exchangers [9,10] and neutral carriers [11–17] as ionophores. Some organic ligands like diamine mercury chloride [18] and poly (4-vinylpyridine) [19] have also been utilized. Although the PVC-based membrane electrodes for mercury(II) have wide linear concentration ranges [11–16], they have narrow pH working ranges and shorter lifetimes [12,13,15–17]. Others suffer from silver(I) interference [11,14,18–20].

Coated wire electrodes (CWE) for Hg(II) ion have been reported [20,21]. The ISE-type CWE is particularly meant to be used in flow injection analysis (FIA) since it is more robust and has no leakage of the internal filling solution while in position in the flowing stream of a flow cell. Only a few flow-through cells are being developed for Hg(II) detection by potentiometric analysis [22,23], mostly with narrow Hg(II) linear concentration ranges of 1 × 10^−5^ to 1 × 10^−3^ M. The FIA technique is a powerful analytical tool for monitoring metal ions in environmental water samples [24]. It has many advantages in terms of reagent consumption, robustness, simplicity of sample and reagent manipulation and fast analysis without risk of sample contamination [24].

In this report a cheap, portable, ease to fabricate and highly selective CWE for mercury(II) has been developed using PVC-thiosemicarbazone as the membrane. The characteristics of the CWE were studied in the conventional way and it was later applied in FIA analysis to determine mercury(II) ion in environmental waters and dental amalgam samples.

## 2. Experimental

### 2.1. Chemicals and Solutions

High molecular weight PVC and potassium tetrakis(4-chlorophenyl) borate (KTClPB) were purchased from Fluka Chemie (Buchs, Switzerland). Mercury(II) nitrate, *o*-nitrophenyloctyl ether (NPOE), dioctyl phthalate (DOP), tributyl phosphate (TBP), bis(2-ethylhexyl) sebacate (BEHS), tris(2-ethylhexyl) phosphate (TEHP), sodium tetraphenylborate (NaTPB), potassium tetrafluoroborate (KBF_4_), oleic acid (OA), tetrahydrofuran (THF), 4-ethyl-3-thiosemicarbazide and 4-dimethylamino-benzaldehyde were obtained from Sigma Aldrich (St. Louis, MO, USA). Tri(dodecyl)methyl ammonium chloride (TDDMACl) was obtained from Acros (Gillman, Australia). All the solutions were prepared from analytical grade chemicals with pure water (18.2 MΩ·cm) from a Millipore (Billerica, MA, USA) Direct Q syste. The water samples were kept in 2.5 L Nalgene high density polyethylene (HDPE) bottles, from Nalgene (Rochester, NY, USA).

**Figure 1.**
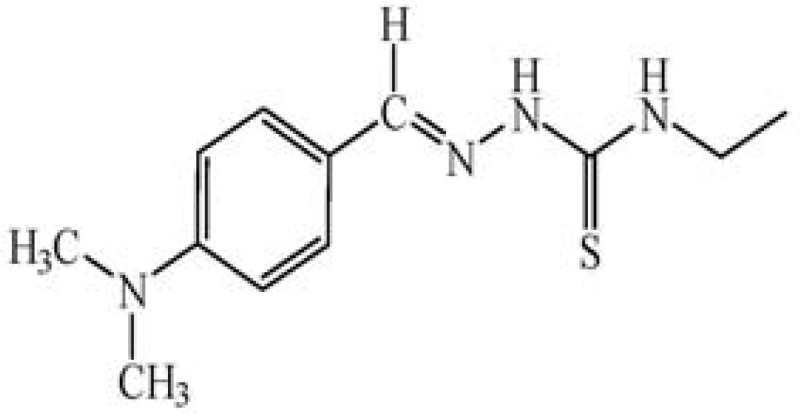
4-(Dimethylamino)benzaldehyde 4-ethylthiosemicarbazone (DMABET).

**Figure 2.**
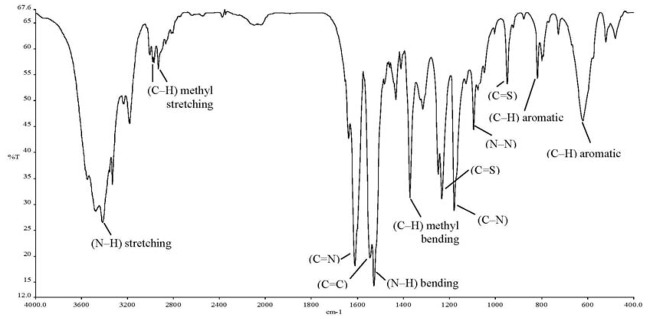
FT-IR spectrum of 4-(dimethylamino)benzaldehyde 4-ethylthiosemicarbazone (DMABET).

The ionophore 4-(dimethyl-amino)benzaldehyde 4-ethylthiosemicarbazone (DMABET, Figure 1) was prepared according to the literature [25] through dropwise addition of 4-dimethylaminobenzaldehyde to 4-ethyl-3-thio-semicarbazide in ethanol. Yellow crystals were obtained after recrystallization from ethanol. Characterization by standard spectroscopic techniques, including FT-IR (Figure 2, Table 1) confirmed the structure.

### 2.2. Instrumentation

A 720A Orion (New Hyde Park, NY, USA) ion meter equipped with a glass electrode and Ag/AgCl reference electrode was used for pH and electromotive force (emf) of mercury(II) ion measurements. A LEO SUPRA 55VD ultra-high resolution field emission scanning electron microscope (FESEM) (Zeiss, Jena, Germany) was used for surface morphology studies. Since the FESEM technique demands a suitable conductive membrane to decrease the charging caused by the electron beam the non-conducting polymeric membrane used was coated with a conducting metal like silver or gold prior to the analysis. A HI 200 M magnetic stirrer (Hanna, Singapore) with a small microbar was used for continuous mixing of solutions during emf measurements.

For the FIA study a multichannel peristaltic pump Miniplus 3 Gilson (Paris, France) was used to propel the solutions through Teflon^®^ tubing (0.8 mm i.d.). A low-pressure four way rotary injection valve model 5020 Rheodyne (Rohnert Park, CA, USA) was used to inject the samples into the flow-through cell. A Model FIP-3 Perspex flow-through cell (3.5–2.5 cm) of the wall-jet design Chemflow Devices (Melbourne, Australia) with an in-built Ag/AgCl reference electrode was utilized. Potentiometric peak responses were obtained on a single chart recorder model BD 111 Kipp and Zonen (Delft, The Netherlands). An A Analyst 200 Perkin Elmer (Waltham, MA, USA) atomic absorption spectrophotometer was used for the mercury cold vapour technique.

### 2.3. Fabrication of Coated Wire Electrode

A number of polymeric membranes based on DMABET were prepared by mixing high molecular weight PVC powder as a polymer matrix, DMABET as an ionophore and different plasticizers ranging from a highly polar one (NPOE) to a low polarity one (TEHP). Various ionic additives were also tested in the membranes to diminish the resistivity of the non-conducting polymer as well as to increase the conductivity of the whole membrane [26]. All these components were dissolved in 2 mL THF with vigorous blending to form a homogenous transparent liquid, then the THF is allowed to slowly evaporate at room temperature until an oily concentrated mixture was obtained. A 2B graphite pencil lead (1.8 mm o.d. and 6 cm length) was immersed in the viscous membrane mixture. The solvent was allowed to evaporate for one hour at room temperature. A thin film of heterogeneous polymeric membrane on the coated graphite electrode is crucial to obtain reproducible responses of the electrode [12]. The fabricated CWE is then kept in a desiccator until further use.

### 2.4. The Emf Measurement

The potential measurements were obtained at 25 ± 2 °C and in 0.1 M KNO_3_ at constant ionic strength. 0.1 M NaNO_3_ was used as ionic strength modifier for measuring the selectivity coefficient toward Hg^2+^ in the presence of K^+^. The following cell scheme was used throughout the study:

Ag,AgCl||sample|membrane|graphite pencil rod

The CWE was conditioned by immersing the membrane part into 1 × 10^−5^ M Hg(NO_3_)_2_ for 1 h before use. A magnetic stirrer was used for continuous stirring of the solution during the measurements. The sample solution was always kept in pH range used by dropwise addition of either concentrated nitric acid or 1 M sodium acetate.

### 2.5. Flow System

**Figure 3.**
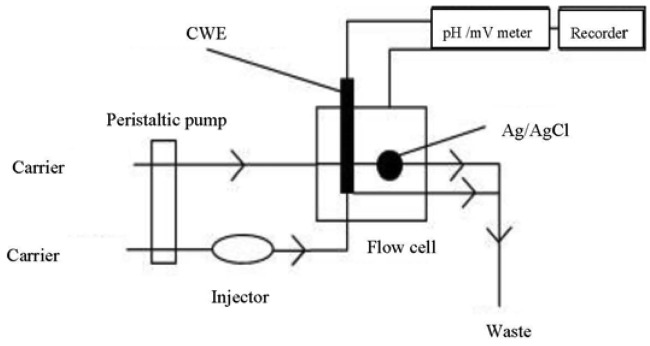
Schematic of the FIA system used.

The carrier stream of 0.1 M KNO_3_ at the pH range 1–5 was propelled into the flow-through cell at a flow rate of 1 mL·min^−1^ using the peristaltic pump (Figure 3). A sampling volume of 85 μL was applied using the designated Teflon^®^ tubing. The CWE was conditioned by propelling 1 × 10^−5^ M Hg(NO_3_)_2_ solution through it for 1 h.

## 3. Results and Discussion

### 3.1. Membrane Characterizations

The ratios of PVC, DMABET, different plasticizers and ionic additives were examined. Although the proportion of the inert PVC bound to the ionophore is important to ensure the physical properties of the membrane like elasticity and mechanical stability, the addition of plasticizer further improves the mobility of the membrane components and gives a homogenous organic phase [26]. Additionally, it may influence the selectivity properties of the membrane.

From Table 2, it is obvious that the incorporation of polar NPOE plasticizer into the PVC-DMABET membrane has reduced its selectivity towards Hg(II), while the less polar BEHS has shown better results. Electrode No. 6 showed a linear concentration range from 1 × 10^−5^ to 0.1 M with almost Nernstian slope and an improved response time. This shows the polarity of plasticizers may influence the equilibrium kinetics of charged species in the solutions [27].

Integrating the ionic additives to the heterogeneous membrane has the advantages of decreasing the anion interferences by expelling them from the membrane phase and reducing the electrical resistance of the membrane [26]. Table 2 also indicates that the addition of KTClPB improved the selectivity and the response time by diminishing the membrane electrical resistance as in the electrode No. 9. However, the amount of the ionic additive introduced in the membrane should be carefully controlled [27]; otherwise it may affect the electrode response as has been illustrated. The optimized heterogeneous membrane of 40 mg PVC:1 mg DMABET:80 mg BEHS:0.7 mg KTClPB was used for further studies.

As the signal of the ISE is pH dependent, the response of the CWE over the pH range 1–8 was investigated. The emf was constant in the pH range of 1–5, but beyond this the emf was decreased. This is probably due to the formation of Hg(OH)^+^ species, which are in equilibrium with Hg(II) and OH^−^ ions. In the acidic medium Hg(II) is the prevalent species, while in nearly neutral media, the most dominant ion is Hg(OH)^+^ [17]. The current membrane based on DMABET ionophore is proven to function over a much wider acidic pH region, whilst other thiosemicarbazone membranes [15] have an inferior pH range. Table 2 also shows that the response time of the electrode No. 6 has been further improved once the ionic additive KTClPB is incorporated into the membrane (see electrode No. 9). The proposed electrode has a response time of 30 s. This is attributed to the formation of Hg^2+^-ionophore-TClPB^−^ ion pairs in the membrane [12], which leads to an increase in the uptake of the Hg(II) from the solution. Additionally, the electrical resistance of the non-conducting polymeric membrane is minimized with the presence of these additives [26]. Investigation on the storage stability and the life- time indicates that the proposed CWE for mercury(II) can be used for at least four months without deviation in its response. During this 120 days period, the slope and linear range did not change and stayed almost constant at 27.8 ± 1 mV·decade^−1^. After 125 day the CWE began to deteriorate and the slope decreased to 23.1 mV. At 140 day the slope was 19.5 mV. The relatively long period of stability is the result of the optimum lipophilicity of the ionophore and plasticizer used [14].

The response of the electrode is the result of exchange mechanism between the Hg(II) ion in solution and those in the membrane at the thiol group of DMABET as in the following equation:

(1) $$\underset{\left(\text{membrane}\right)}{\emptyset -{\text{S}}^{2-}}\underset{\left(\text{solution}\right)}{-{\text{Hg}}^{2+}+\underline{{\text{Hg}}^{2+}}}\underset{\left(\text{membrane}\right)}{↔\emptyset -{\text{S}}^{2-}}\underset{\left(\text{solution}\right)}{-\underline{{\text{Hg}}^{2+}}+{\text{Hg}}^{2+}}$$

where Ø is DMABET

Based on hard-soft acid-base theory the bond formation between mercury and ionophore most likely occurs at the thiol group. Since both are categorized as soft it is expected that the complex formed would be a stable one. However, in this study the size of the ionophore used is much bigger, hence providing facile exchange of counter ions between the membrane phase and solution phase (Donnan exchange) and at equilibrium generating the emf response. The permselectivity of the membrane ensures that no significant amount of the counter ions may enters the membrane phase, *i.e.*, the so called Donnan exclusion [26]. This exclusion of the undesired ions can be achieved with electrically neutral carriers and also the addition of the lipophilic ion salts.

### 3.2. FESEM Study

The Nernstian behavior of an ISE depends on its surface characteristic. FESEM with ultra-high resolution analysis is used to detect the bulk surface morphology of the membrane without pretreatment. In this study, three different membranes were prepared as follows: (a) 40 mg PVC:1 mg DMABET, (b) 40 mg PVC:1 mg DMABET:80 mg BEHS and (c) 40 mg PVC:1 mg DMABET:80 mg BEHS:0.7 mg KTClPB, according to the standard procedure in the Experimental part.

**Figure 4.**
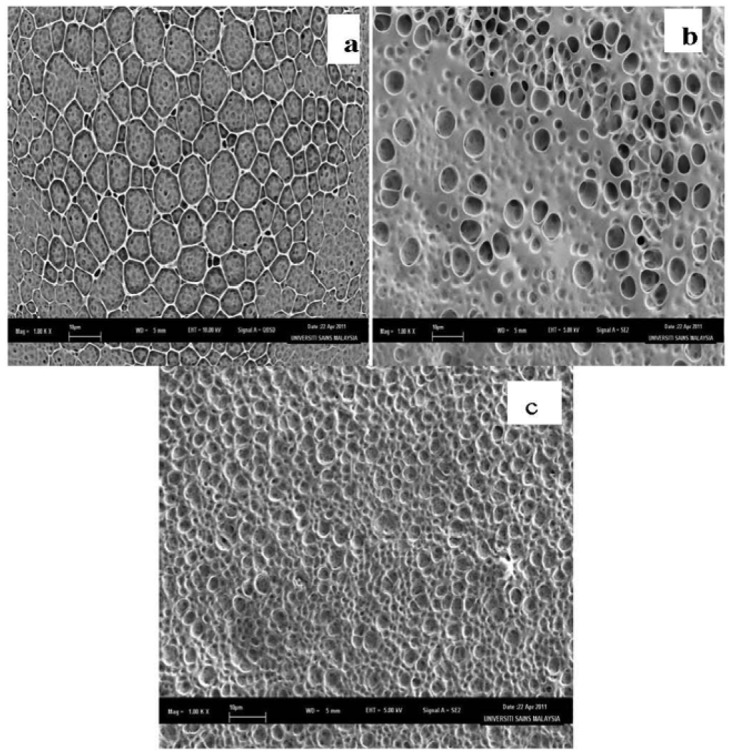
FESEM of (**a**) 40 mg PVC:1 mg DMABET; (**b**) 40 mg PVC:1 mg DMABET:80 mg BEHS and (**c**) 40 mg PVC:1 mg DMABET:80 mg BEHS:0.7 mg KTClPB membranes (1000× magnification).

Figure 4 shows the FESEM results for the three membranes which confirm significant improvement in the membrane properties with the addition of plasticizer and ionic additive. Figure 4(a) shows that the polymer matrix without additives displayed asymmetrical pores within the membrane. The graphite electrode coated with this membrane has shown non-Nernstian behavior. This is probably due to the absence of the plasticizer which may affect the mobility of the membrane components [26]. Besides, the presence of the plasticizer may influence in the equilibrium kinetics of the charged species in the solution and thus the selectivity of the membrane towards the target ion, *i.e.*, Hg(II) [27]. Figure 4(b) shows a membrane of PVC-DMABET and BEHS plasticizer with elastic smoother surface and less porosity. Figure 4(c) corresponds to the membrane of PVC, DMABET, BEHS plus the ionic additive KTClPB, which shows a more homogenous surface with disappearing pores. These membranes (Figure 4(b,c)) have Nernstian response. The addition of the lipophilic ionic additives salts will further improve the response of the electrode and their slope due to the diminishing of the membrane electrical resistance depending on Donnan exchange equilibrium [26]. These ionic additives expel the anions from the membrane surface, so that the interferences of the counter ions are excluded and thus increasing the selectivity. Besides, the addition of additives will increase the plasticity and material fluidity of the membrane constituents, thus escalate the electrode selectivity [26].

### 3.3. Selectivity and Interferences

The selectivity coefficient (K^p^°^t^_Hg,M_) describes the ability of an ISE to prefer analytes than the interferents. It is evaluated by the emf response of the ISE in mixed solutions of the analyte/primary ion, Hg(II) and interference/secondary ion, M. The membrane of an ISE is responsible for the emf response and selectivity of the entire electrode. The predominant factor that governs the membrane selectivity is the distribution of the cation complexes. Regardless of the ligand structure and cation size the complex formation is the determining factor for selectivity. In order to examine the selectivity of the optimized mercury(II) CWE, the fixed primary ion method (FPIM) was used to measure the values of K^p^°^t^_Hg,M_ for different cations and anions interferences [19]. In this study, the concentration of Hg(II) was kept at 1 × 10^−4^ M, while the interference concentrations were varied between 1 × 10^−6^ and 0.1 M, prepared together at constant ionic strength of 0.1 M KNO_3_ except for K(I) ion where a constant ionic strength of 0.1 M NaNO_3_ was used. All the measurements were achieved with standard deviations of three readings (n = 3). Previous mercury(II)-ISEs have suffered from interference by Ag(I) [11,13–15,18–21], Fe(III) [13] and Cd(II) [18] ions.

From Table 3, it is obvious that these ions do not interfere with the response of the proposed CWE to mercury(II) ion, neither did the anions. The mercury(II) CWE built has shown high selectivity towards Hg(II) over many alkalis, alkaline earths, heavy and transition metal ions. But more interesting is the good selectivity of the electrode towards Hg(II) over Ag(I). Although Hg(II) and Ag(I) are both soft acid but Hg(II) ion shows stronger affinity for ligands with S and N atoms as in the thiosemicarbazone resulting in the formation of stable complexes [15]. The reaction of thiosemicarbazone derivatives with Hg(II) has, however, formed labile complexes through mercury(II)-thiol bond [28].

### 3.4. FIA Optimization

It can be seen that the electrochemical parameters obtained for the proposed mercury(II) CWE show that DMABET makes an outstanding ionophore for determination of Hg(II) ion. Because of this the developed CWE is applied in an FIA system due to their attractive advantages [22].

The sensitivity, selectivity and peak heights of a CWE in FIA system depend significantly on the membrane composition, specifically plasticizers [23]. However, the use of plasticized PVC membranes in a CWE has some disadvantages. The gradual leaching of the plasticizer into the sample solution deteriorates the signal to noise ratio (S/N) and the slope of the electrode, thus limiting the endurance *i.e.*, lifetime of the membrane and the usage of the electrode. However, in this study the response of the proposed plasticized mercury(II) CWE is good and remains stable for almost four months.

**Figure 5.**
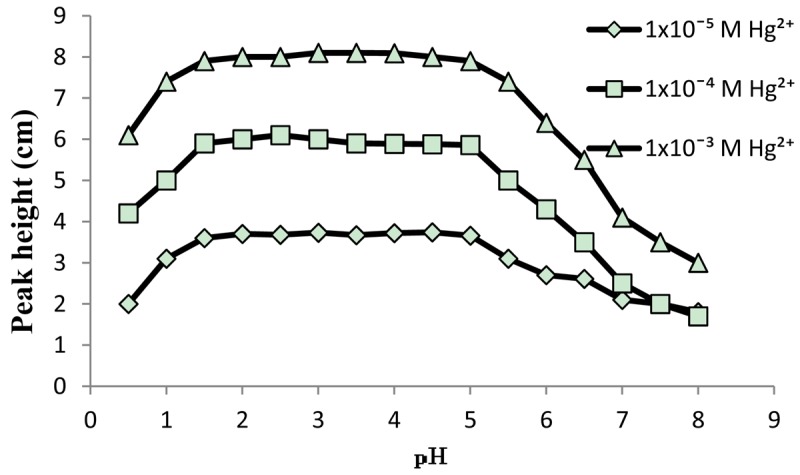
pH profile for mercury(II)-ISE in a FIA system.

A solution of 0.1 M KNO_3_ in the pH range 1–5 was used as carrier to get stable baseline for standard Hg(NO_3_)_2_ solutions with concentration range of 1 × 10^−6^ to 0.1 M. The effects of sample volume and carrier flow-rate on the signal (as peak height) were studied by injecting 1 × 10^−3^, 1 × 10^−4^ and 1 × 10^−5^ M Hg(II) into the FIA system. The utilization of bigger sample volume and lower flow rates has enhanced the response. The optimum values obtained for flow rate and sample volume were 1 mL·min^−1^ and 85 μL, respectively. The pH profile of the electrode in the pH range of 0.5–8 in the concentrations 1 × 10^−3^, 1 × 10^−4^ and 1 × 10^−5^ M Hg(II) is shown in Figure 5. The peak heights of FIA outputs obtained from injecting standard solutions of Hg(II) were remain stable in the pH range of 1–5. At pH 5.5 and higher the peaks are lowered. The lowering in response is due to competition between Hg(II) and OH^−^ to the site in the PVC-DMABET membrane.

### 3.5. Linear Range and Sensitivity

**Figure 6.**
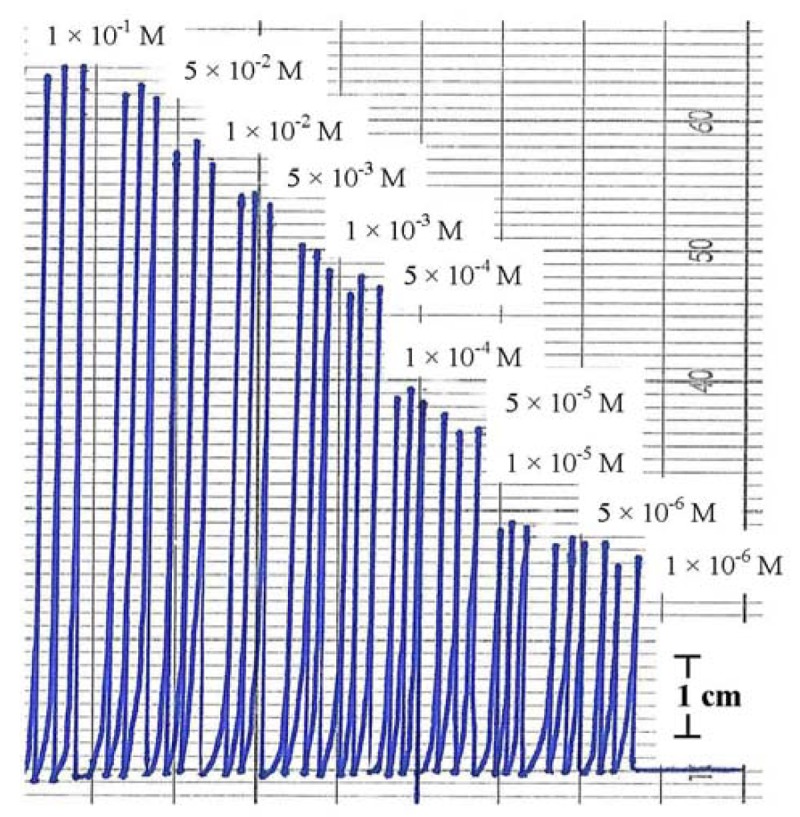
The FIA peak heights for the standard solutions of Hg(NO_3_)_2_ in triplicate measurements.

Injecting the standard solutions of Hg(NO_3_)_2_ in the concentration range of 1 × 10^−6^–0.1 M to the FIA system showed good reproducibility either from low to high concentration or vice versa. Figure 6 shows the FIA signals of Hg(II) standard solutions where each concentration has been injected three times to the flow system. The linear range was 5 × 10^−6^–0.1 M with regression linear coefficient (R^2^) 0.984 and detection limit (DL) 5 × 10^−6^ M.

Although other state of the art instruments provide better linear range and Hg(II) detection limit, with the low cost, possibility of miniaturization and robustness of CWE and FIA for determination of mercury(II) seems to enhance its utility. Although the detection limit of other Hg(II) ISE in FIA systems [23,29] are relatively the same as that obtained with the proposed mercury(II) ISE, the latter has a wider linear range (Table 4).

### 3.6. Analysis of Hg(II) in Environmental Waters and Dental Amalgam Samples

The FIA system has been successfully applied for determination of Hg(II) in amalgam and different water samples. Environmental surface water samples were collected using a plastic scoop and then stored in HDPE bottles. Nitrous acid (5 mL) was added to the water samples to avoid improper precipitation of mercury prior to storage at 4 °C.

For analysis of mercury in the dental amalgam sample 1.0 g of the amalgam sample was dissolved in a minimum amount of concentrated nitric acid with heating [21]. The dissolved sample was further diluted to 100 mL using triple distilled water.

The direct determination method (DDM) was employed in the FIA system for estimation of mercury in the dental amalgam and water samples. DDM and cold vapour AAS results were compared and have been found to be in good agreement with each other with recovery almost 100% and high reproducibility (RSD < 1%) (Table 5).

**Figure 7.**
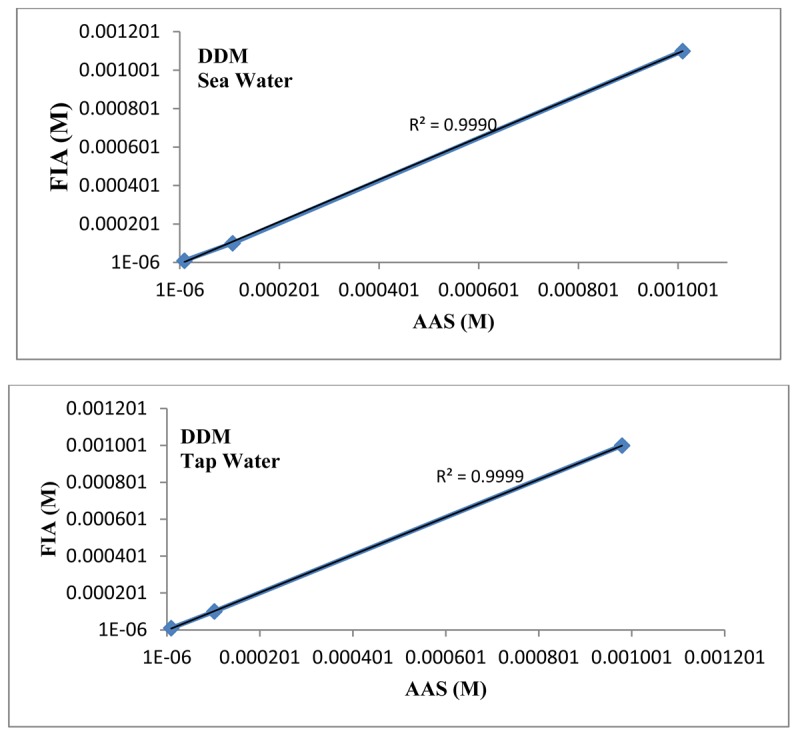
Validation of the proposed mercury(II)-ISE in FIA system by AAS method.

The linear plot between the proposed mercury(II) ISE and the AAS results (Figure 7) has a correlation coefficient (R^2^) of 0.9999 indicating that the proposed mercury(II) electrode can be efficiently used in FIA system for determination of Hg(II) in natural and environmental samples.

A sampling frequency of 30 samples·h^−1^ was obtained with the developed system. The comparison between the accuracy of the two methods was performed at the 95% confidence level and 5 degree of freedom using a t-test: t_table_(95%)_F=5_ = 2.57 > t_calculated_ = 1.11. This indicates that there is no significant difference between both methods.

## 4. Conclusions

This work revealed that the proposed CWE constructed from PVC, DMABET and various kinds of plasticizers and additives can be an excellent ISE for mercury(II). The sensor was found to have Nernstian response in the pH range 1–5. It presents a good selectivity towards Hg(II) ion over most cations, especially Ag(I). The device has been applied in a FIA system for the determination of mercury(II) in environmental water samples and dental amalgam with results comparable to the standard method.

## Figures and Tables

**Table 1.** Summary of FT-IR analysis of DMABET.

**Absorption band**	**Frequency (cm^−1^)**	**(Intensity) Vibration**
*v* (C=N)	1,610	(str, sh) stretching
*v* (C=C) aromatic	1,545	(m, sh) stretching
*v* (C–H) aromatic	817	(m, sh) bending
	620	(m, br) bending
*v* (C–N)	1,179	(str, sh) stretching
*v* (N–N)	1,094	(m, sh) stretching
*v* (N–H)	3,416	(m, br) stretching
	1,527	(str, sh) bending
*v* (C=S)	1,232	(str, sh) stretching
	948	(str, sh) stretching
*v* (C–H) methyl	2,968, 2,931	(w) stretching
	1,371	(str, sh) bending

**Table 2.** Optimization for PVC-DMABET membrane composition 1.

**Electrode No.**	**PVC (mg)**	**DMABET (mg)**	**Plasticizer (mg)**	**Ionic additives (mg)**	**LR (M)**	**Slope (mV per decade)**	**pH range**	**DL (M)**	**RT (s)**
1	40	1	0	0	5 × 10^−5^–0.1	37 ± 6	1–5	5 × 10^−6^	49–60
2	40	1	80 (TBP)	0	5 × 10^−5^–0.1	37 ± 4	1–5	1 × 10^−4^	34–60
3	40	1	80 (NPOE)	0	5 × 10^−5^–0.1	38 ± 5	1–5	1 × 10^−5^	74–92
4	40	1	80 (TEHP)	0	5 × 10^−5^–0.1	36 ± 4	2–5	1 × 10^−5^	74–120
5	40	1	80 (DOP)	0	5 × 10^−5^–0.1	32 ± 3	1–3	1 × 10^−5^	106–120
6	40	1	80 (BEHS)	0	1 × 10^−5^–0.1	31 ± 1	1–5	5 × 10^−6^	40–60
7	40	1	80 (BEHS)	0.7 (NaTPB)	1 × 10^−5^–0.1	23.2 ± 1	1–5	5 × 10^−6^	33
8	40	1	80 (BEHS)	0.7 (KBF_4_)	1 × 10^−5^–1 × 10^−2^	30 ± 1	1–5	5 × 10^−6^	36
9	40	1	80 (BEHS)	0.7 (KTClPB)	1 × 10^−5^–0.1	27.8 ± 1	1–5	5 × 10^−6^	30
10	40	1	80 (BEHS)	0.7 (TDDMACl)	1 × 10^−5^–0.1	25.5 ± 1	1–5	5 × 10^−6^	38
11	40	1	80 (BEHS)	0.7 (OA)	1 × 10^−5^–0.1	37.3 ± 1	1–5	5 × 10^−6^	38
12	40	1	80 (BEHS)	0.5 (KTClPB)	1 × 10^−5^–0.1	33 ± 1	1–5	5 × 10^−6^	30
13	40	1	80 (BEHS)	0.9 (KTClPB)	1 × 10^−5^–0.1	34 ± 1	1–5	5 × 10^−6^	30
14	40	1	80 (BEHS)	1.3 KTClPB)	1 × 10^−5^–0.1	34 ± 1	1–5	5 × 10^−6^	30
15	40	1	80 (BEHS)	1.7 (KTClPB)	1 × 10^−5^–0.1	40.6 ± 1	1–5	5 × 10^−6^	30

LR: linear range; DL: detection limit; RT: response time.

1 The standard deviations of the data with plus minus are based on triplicate analyses.

**Table 3.** Selectivity coefficients of various interfering cations and anions for electrode (No. 9), (n = 3).

**Cations**	**K^pot^_Hg,M_**	**Anions**	**K^pot^_Hg,M_**
Ag^+^	5.69 (±0.04) × 10^−3^	Cl^−^	1.83 (±0.02) × 10^−7^
Cu^2+^	1.00 (±0.02) × 10^−3^	NO_3_^−^	2.10 (±0.03) × 10^−9^
Pb^2+^	9.71 (±0.03) × 10^−4^	HCO_3_^−^	6.86 (±0.03) × 10^−8^
Cd^2+^	9.10 (±0.02) × 10^−4^	SO_4_^2−^	3.60 (±0.03) × 10^−7^
Zn^2+^	1.00 (±0.03) × 10^−3^	HPO_4_^2−^	3.58 (±0.03) × 10^−7^
Ca^2+^	7.71 (±0.02) × 10^−4^		
Mg^2+^	5.00 (±0.031) × 10^−4^		
Fe^3+^	1.23 (±0.04) × 10^−3^		
Ni^2+^	1.10 (±0.02) × 10^−3^		
Co^2+^	1.00 (±0.03) × 10^−3^		
Al^3+^	4.00 (±0.02) × 10^−4^		
K^+^	3.40 (±0.02) × 10^−4^		
Na^+^	3.00 (±0.02) × 10^−4^		

**Table 4.** The performances of mercury(II) ISEs in FIA systems.

**Ionphore of mercury(II) electrodes in FIA system**	**LR (M)**	**DL (M)**
4,7,13,16-tetrathenoyl-1,10-dioxa-4,7,13, 16-tetraazacyclooctadecane [23]	3.16 × 10^−6^–1 × 10^−3^	3.16 × 10^−6^
7,16-dithenyl-l,4,10,13-tetraoxa-7, 16-diazacyclooctadecane [28]	3.16 × 10^−6^–1 × 10^−3^	3.16 × 10^−6^
[This study]	5 × 10^−6^–1 × 10^−1^	5 × 10^−6^

**Table 5.** Determination of Hg(II) ion in dental amalgam and water samples using DDM in FIA and cold vapour AAS (n = 3).

**Sample**	**Hg(II) added (M)**	**FIA (M)**	**Cold vapour AAS a (M)**	**Recovery (%)**
Sea water	1.0 × 10^−5^	1.03 (±0.31) × 10^−5^	1.01 (±0.11) × 10^−5^	103
	1.0 × 10^−4^	1.07 (±0.36) × 10^−4^	1.01 (±0.09) × 10^−4^	107
	1.0 × 10^−3^	1.01 (±0.25) × 10^−3^	1.1 (±0.1) × 10^−3^	101
Tap water	1.0 × 10^−5^	1.02 (±0.30) × 10^−5^	1.05 (±0.13) × 10^−5^	102
	1.0 × 10^−4^	1.03 (±0.36) × 10^−4^	1.01 (±0.12) × 10^−4^	103
	1.0 × 10^−3^	0.98 (±0.24) × 10^−3^	1.0 (±0.1) × 10^−3^	98
Dental amalgam (%)		55.62 (±0.29)	55.10 (±0.10)	

a Standard method was used;

b Values in parentheses are SDs based on three replicate analyses.
